# Synthesis of a Dual-Labeled Probe of Dimethyl Lithospermate B with Photochemical and Fluorescent Properties

**DOI:** 10.3390/molecules16129886

**Published:** 2011-11-28

**Authors:** Eunyoung Lim, Jeremy Ricci, Mankil Jung

**Affiliations:** Department of Chemistry, Yonsei University, Seoul 120-749, Korea

**Keywords:** dimethyl lithospermate B, target identification, photochemical and fluorescent probe, dual probe

## Abstract

Dimethyl lithosermate B (DLB) is a highly potent natural antioxidant and antidiabetic polyphenol with unknown mode of action. To determine its cellular targets, a photochemical and fluorescent dimethyl lithopermate B probe was designed and efficiently synthesized. The dual-labeled chemical probe for biological application was evaluated by UV and fluorescence to determine its electrochemical absorption and emission properties. This probe could be valuable for investigating ligand-protein interactions and subcellular localization.

## 1. Introduction

Lithospermic acid B (LAB, **1**), a recently isolated component of *Salvia miltiorrhiza*, is known to have multiple pharmacological activities, including: (1) hepatoprotection [1]; (2) endothelium-dependent vasodilation [2]; (3) the ability to lower blood pressure in hypertensive rats [3]; and (4) amelioration of cephaloridine, adenine-, and ischemia/reperfusion-induced renal injury in rats [4,5]. In addition dimethyl lithospermate B (DLB, **2**), a minor component of the root extract from *Salvia miltiorrhiza*, has been shown to be a Na^+^ channel agonist [6] and a suppressor of arrhythmogenesis [7]. As a result, there is great interest in the therapeutic potentials of LAB and dimethyl LAB in atherosclerosis and restenosis.

Previously, we reported an effective method for isolating magnesium lithospermate B from *Salvia miltiorrhiza* [8] and found that magnesium lithospermate B, a potent antioxidant, had protective effects on diabetes-induced renal disease [9] and vascular injury response [10]. Despite its various biological activities, the specific mechanism of action of LAB in atherosclerosis and restenosis remains unknown. This has led to the design of chemical probes for the study of LAB-binding proteins, which can provide a useful method for direct probing to define target proteins. Photoaffinity labeling using a photolabile probe is particularly useful because of the ability of the probe to photochemically form a covalent adduct with the target protein. The diazirine photophores have gained popularity over other photoreactive groups due to their highly desirable properties as photoaffinity probes. In particular, at around 360 nm, diazirines undergo rapid photoactivation into highly reactive carbene species that can even form carbon–carbon covalent bonds with aliphatic hydrocarbons [11,12]. Furthermore, they can be kept small enough that the modified ligand does not lose its biological activity [13].

In order to identify the photoaffinity labeling products after binding and photolysis, a reporter group should be contained within the photophore or somewhere else in the ligand [14]. Radioisotopes have been employed for this purpose. As an alternative to inconvenient radioisotopes, photoaffinity biotinylation can be used. However, the polarity and large size of the biotin-anchored tag often render the ligand-affinity conjugate substantially less active than the parent ligand [13]. In order to circumvent these shortcomings, dual probes have been proposed that carry a fluorescent group for imaging the binding of target protein after photoaffinity labeling. Thus, dual probes may further enhance the efficiency of identifying target proteins of LAB. In the literature, fluorescent derivatives of natural products have been employed as probes to identify subcellular localization upon labeling with the probe. A small-sized dansyl group as a fluorescence probe with moderate lipophilicity is one of the most common labels used in biological applications [15,16].

The rationale for attaching the labels to the phenol group in the benzofuran part of DLB is based on the fact that the *o*-dihydroxy groups in LAB are the most essential moiety for antioxidant activity due to possible formation of a stable phenoxy radical based on delocalizing electron movement [17,18]. Thus, modification of the phenol moiety at benzofuran part does not inactivate the biological activity of DLB significantly, compared to the three *o*-dihydroxy groups.

**Figure 1:**
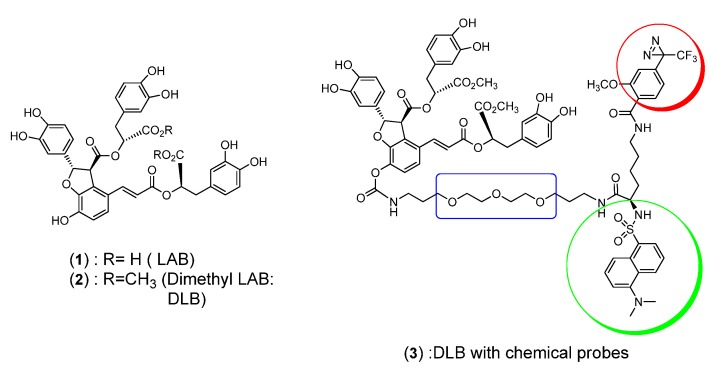
DLB (**2**) and its chemical and fluorescent probe **3**.

Based on the above considerations, we synthesized and performed a photochemical and fluorescent evaluation of a bifunctional dimethyl LAB derivative **3** with a diazirine moiety as the photoactivable group and a dansyl moiety as the fluorescence probe (Figure 1).

## 2. Results and Discussion

### 2.1. Synthesis of DLB Photoaffinity Probe

**Scheme 1:**
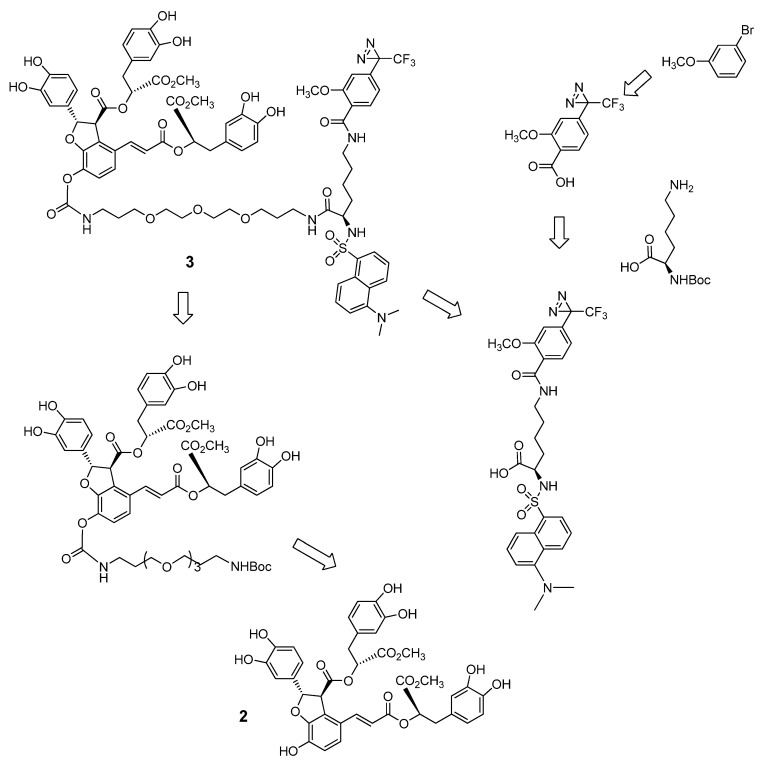
Restrosynthetic analysis for chemical probe of DLB **3**.

The retrosynthetic analysis and strategy for preparation of difunctional chemical probe **3** are shown in Scheme 1. In the procedure, a diazirine moiety is incorporated as a photoreactive group into the probe for the target protein and a dansyl group is inserted as a fluorophore to detect and quantify the target protein.

**Scheme 2:**
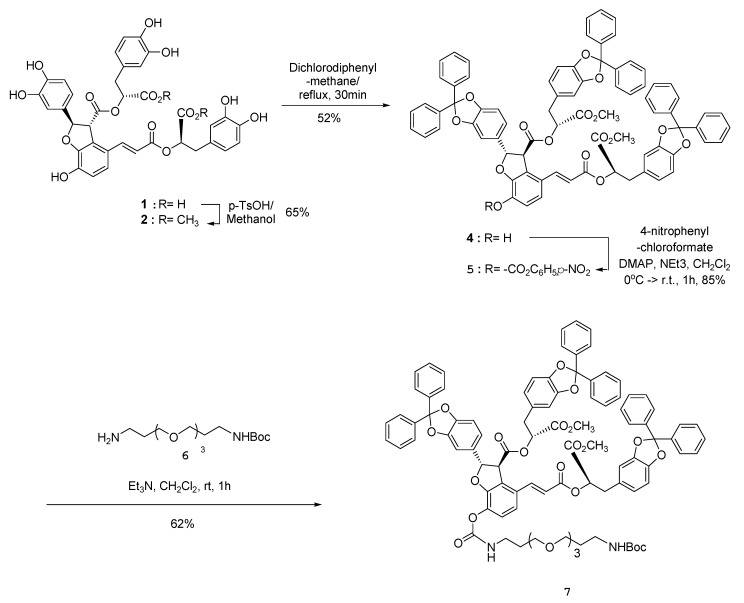
Synthesis of DLB key intermediate **7**.

The natural product LAB (**1**) was isolated as described previously [8]. The synthesis of the protected key fragment **5** is outlined in Scheme 2. LAB (**1**) was easily converted to dimethyl LAB (**2**) using *p*-toluenesulfonic acid as a catalyst in methanol [7]. Protection of all catechol groups of compound **2** with dichlorodiphenylmethane and heating at 150 °C for 30 min [19] resulted in compound **4**. The resulting intact benzofuran hydroxyl group of **4** was converted into the 4-nitrophenyl carbonate **5** in 85% yield. The ethylene glycol linker **6** [20] was introduced to the key intermediate **5** by substitution of the 4-nitorophenyl carbonate group to produce compound **7** (Scheme 2).

**Scheme 3:**
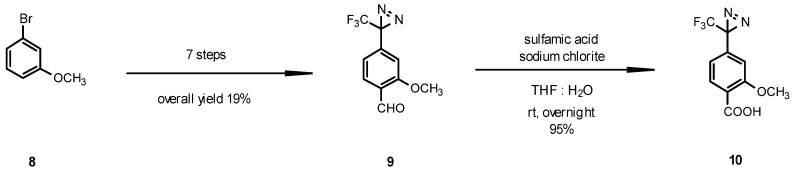
Synthesis of diazirine acid **10**.

The key intermediate diazirine aldehyde **9** was prepared in seven steps starting from 3-bromoanisole (**8**) as described by the previous report for introduction of the 3-(trifluoromethyl)-diazirin-3-yl group [21,22]. The final oxidation of the aldehyde **9** to the acid **10** was performed using the combination of sodium chlorite with sulfamic acid in THF and water (Scheme 3) [13].

**Scheme 4:**
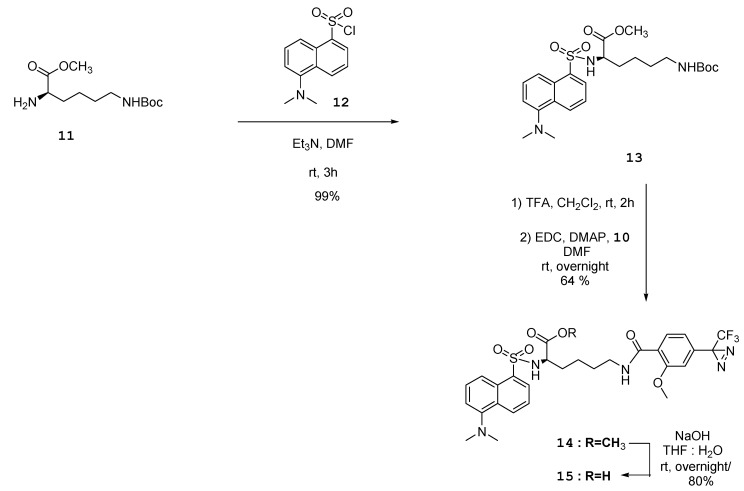
Synthesis of dual-functional group **15**.

**Scheme 5:**
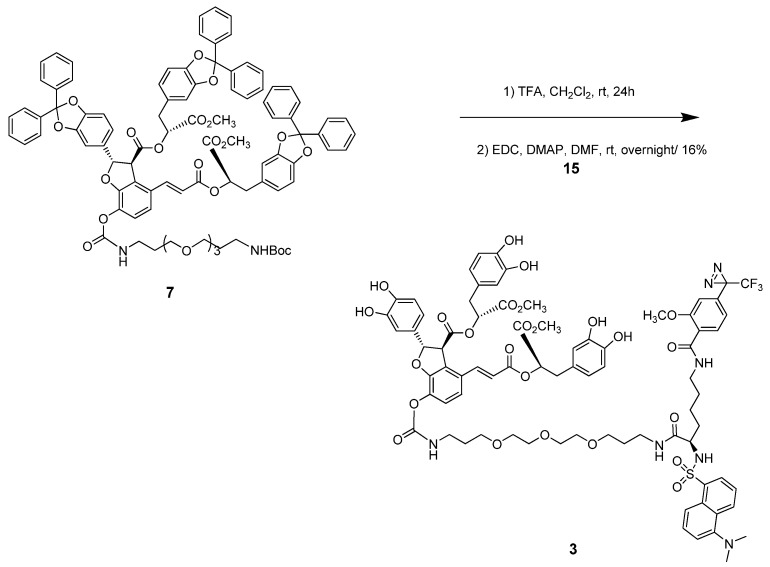
Synthesis of dual chemical probe **3**.

Lysine was chosen to connect LAB with the dansyl group and diazirine moiety as a trifunctional linker. Coupling of dansyl chloride **12** with protected lysine **11** as a starting material under basic conditions resulted in sulfonamide compound **13**. Deprotection of the *t*-Boc group of compound **13** using TFA/CH_2_Cl_2_, followed by amide coupling with diazirine acid **10** using EDC and DMAP afforded compound **14**. The ester group of compound **14** was hydrolyzed using aqueous base to produce diazirine-linked dansyl acid **15** (Scheme 4). The one step deprotection of both the diphenyl and *t*-Boc groups of compound **7** using TFA/CH_2_Cl_2_ followed by amide coupling with the acid **15** with EDC and DMAP in DMF afforded the final dual probe compound **3** in 16% yield (Scheme 5).

### 2.2. Photochemical and Fluorescent Evaluation

In order to obtain parameters for monitoring of the photochemical reaction, as well as the fluorescence properties, we measured UV and fluorescence in aqueous solution for biological application. The photochemical reactions of chemical probe **3** were examined to confirm its reactivity under photoactivation. As shown in other reports, 3-trifluoromethyl-3-aryldiazirine has a characteristic absorbance peak at about 348 nm [23,24,25]. The photochemical kinetics of compound **3** in methanol at excitation wavelength 348 nm for fixed periods of time were measured spectrophotometrically (Supporting Information Figure 1) Since UV absorption of the dansyl group and benzene group of compound **3** is stronger than that of diazirine compound, there was no significant change in absorption spectra.

**Scheme 6:**
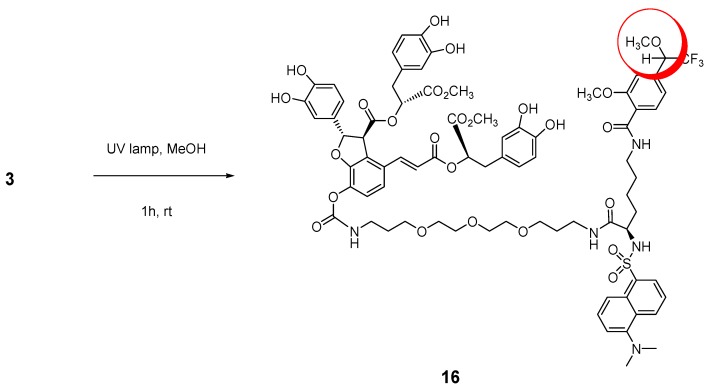
Carbene-trapped adduct 16 by UV irradiation at 365 nm.

Nevertheless, the carbene generated from the diazirine moiety in probe **3** with UV irradiation at 365 nm for 1 h was trapped by CH_3_OH via insertion and the methanol adduct **16** as shown in Scheme 6 was confirmed by UPLC-Q-TOF Mass analyzer (calcd. 1622.5283 for C_78_H_88_F_3_N_5_O_26_SNa[M+Na]^+^, found: 1622.8319). This confirmed carbene generation from the diazirine group in chemical probe **3** by the UV laser beam. Thus, the photoaffinity probe **3** can be transformed into a highly reactive carbene that can remove a hydrogen atom present in its close environment within the target protein-binding site.

**Figure 2:**
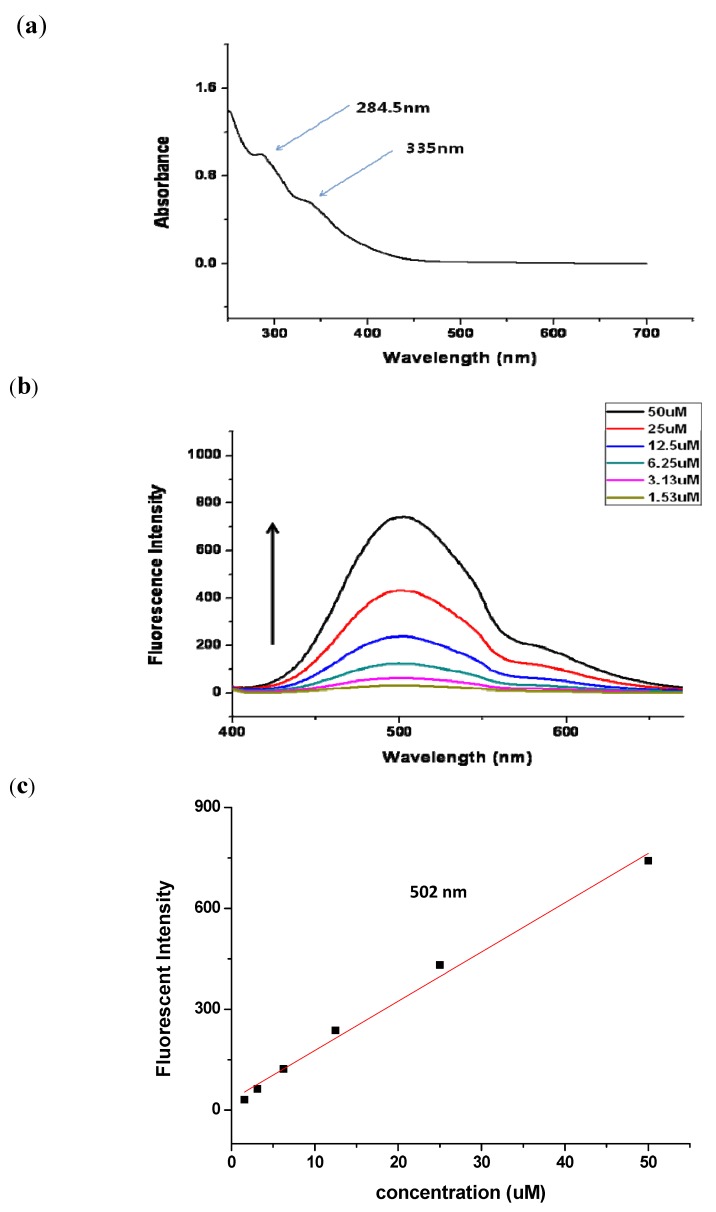
Electronic absorption and emission spectra of dual-functional DLB probe **3**. (**a**) UV spectra in 50 µM aqueous solution; (**b**) fluorescence spectra at 335 nm; (**c**) calibration curve of fluorescence at 502 nm.

The electronic absorption and emission spectra of chemical probe **3** are depicted in Figure 2. Since the maximum effective concentration for biological evaluation for LAB is 50 µM, UV adsorption spectra and fluorescent emission spectra were measured in 50 µM solution in water (0.5% DMSO) at non-toxic and effective cellular level. The compound in water shows absorption peak maxima at 284.5 nm and 335 nm (Figure 2a). The electronic emission spectra of the chemical probe in various concentrations (50 µM to 1.56 µM) are described in Figure 2b. Upon excitation at 335 nm, aqueous solution of **3** shows an emission at 502 nm at 50 µM (peak maximum). The calibration of fluorescence to the various concentrations (50 µM to 1.56 µM) gave a linear range as shown in Figure 2c and thus, the probe could be applied to quantify the target protein. These data will be helpful for cellular localization for target identification studies. Therefore, this dual-labeled chemical probe **3** could be valuable as a bioprobe for investigating ligand-protein interactions.

## 3. Experimental

### 3.1. General

All commercial reagents and solvents were used as received without further purification unless specified. Reaction solvents were distilled from calcium hydride for dichloromethane and from sodium metal and benzophenone for tetrahydrofuran. The reactions were monitored and the *R_f_* values determined using analytical thin layer chromatography (TLC) with Merck silica gel 60 and F-254 precoated plates (0.25-mm thickness). Spots on the TLC plates were visualized using ultraviolet light (254 nm) and a basic potassium permanganate solution or cerium sulfate/ammonium dimolybdate/sulfuric acid solution followed by heating on a hot plate. Flash column chromatography was performed with Merck silica gel 60 (230–400 mesh). ^1^H-NMR spectra were recorded on Bruker DPX-250 or Bruker DPX-500 spectrometers. Proton chemical shifts are reported in ppm (δ) relative to internal tetramethylsilane (TMS, δ 0.00) or with the solvent reference relative to TMS employed as the internal standard (CDCl_3_, δ 7.26 ppm; d_4_-CD_3_OD, δ 3.31 ppm). Data were reported as the chemical shift {multiplicity [singlet (s), doublet (d), triplet (t), quartet (q), and multiplet (m)] coupling constants [Hz], integration}. ^13^C-NMR spectra were recorded on Bruker DPX-250 (63 MHz) or Bruker DPX-500 (125 MHz) spectrometers with complete proton decoupling. Carbon chemical shifts were reported in ppm (δ) relative to TMS with the respective solvent resonance as the internal standard (CDCl_3_, δ 77.0 ppm; d_4_-CD_3_OD, δ 49.0 ppm). Infrared (IR) spectra were recorded on a Nicolet Model Impact FT-IR 400 spectrometer. Data were reported in wave numbers (cm^−1^). The UV-vis spectra were recorded on a Hewlett-Packard HP8453. Fluorescence emission spectra were obtained using a Hitachi F-4500 spectrofluorimeter linked to a Pentium PC running SpectraCalc software package. The slit width was 5.0 nm for both excitation and emission. The photon multiplier voltage was 400 V. UPLC-Q-TOF Mass Spectrometer were recorded on a Micromass Q-TOF ACQUITY UPLC-Mass SYSTEM. MALDI-TOF masses were recorded on an Applied Biosystems 4700 proteomics analyzer spectrometer and high-resolution mass spectrometer (HRMS) analyses were recorded on a JEOL JMS-700 spectrometer.

### 3.2. (2S,3S)-(R)-3-(2,2-Diphenylbenzo[d][1,3]dioxol-5-yl)-1-methoxy-1-oxopropan-2-yl)2-(2,2-diphenylbenzo[d][1,3]dioxol-5-yl)-4-((E)-3-((R)-3-(2,2-diphenylbenzo[d][1,3]dioxol-5-yl)-1-methoxy-1-oxopropan-2-yloxy)-3-oxoprop-1-enyl)-7-hydroxy-2,3-dihydrobenzofuran-3-carboxylate ***(4)***

Dimethyl lithospermate B (**2**, 1 g, 1.34 mmol) and dichlorodiphenylmethane (4 equiv., 1.02 mL, 5.36 mmol) were mechanically mixed, then heated at 150 °C for 30 min. The crude resulting mixture was purified by flash column chromatography using hexane/EtOAc (3/2) as eluent to produce compound **4** (2.92 g, 52% yield). Light yellow solid; [α]_D_^20^ = +98 (c 0.75, CHCl_3_); IR ν_max_ (KBr, CHCl_3_) 3420.14, 3063.37, 3031.07 2953.45, 2927.9, 2849.79, 1955.95, 1887, 1746.23, 1614.13, 1589.54, 1495.04, 1450.69, 1370.18, 1296.41, 1252.54, 1211.08, 1179.26, 1154.19, 1077.05, 1042.34 1022.099 947.359, 919.3978 813.813, 759.334; ^1^H-NMR (250 MHz, CDCl_3_) δ ppm 3.0 (m, 4H), 3.6 (s, 6H), 4.4 (d, *J* = 4.1 Hz, 1H), 5.2 (m, 2H), 5.6 (s, 1H), 5.9 (d, *J* = 4.1 Hz, 1H), 6.2 (d, *J* = 15.8 Hz, 1H), 6.4 (d, *J* = 7.9 Hz, 1H), 6.5 (d, *J* = 1.6 Hz, 1H), 6.7 (m, 8H), 6.9 (d, *J* = 8.5 Hz, 1H), 7.24–7.42 (m, 18H), 7.47–7.69 (m, 13H); ^13^C-NMR (63 MHz, CDCl_3_) δ 36.71, 37.37, 52.30, 52.49, 56.43, 73.32, 74.05, 87.33, 105.77, 108.47, 109.86, 116.27, 116.85, 117.39, 119.35, 121.48, 122.72, 124.28, 126.30, 126.35, 126.40, 128.30, 128.37, 129.14, 129.26, 129.84, 133.92, 140.08, 140.33, 140.44, 146.20, 146.29, 147.09, 147.25, 147.36, 147.62, 147.75, 166.17, 169.40, 170.06, 170.38; MALD-TOF-MS calcd. 1261.3617 for C_77_H_58_O_16_ [M+Na]^+^, found: 1261.2347.

### 3.3. (2S,3S)-(R)-3-(2,2-Diphenylbenzo[d][1,3]dioxol-5-yl)-1-methoxy-1-oxopropan-2-yl) 2-(2,2-diphenyl benzo[d][1,3]dioxol-5-yl)-4-(E)-3-(R)-3-(2,2-diphenylbenzo[d][1,3]dioxol-5-yl)-1-methoxy-1-oxopropan-2-yloxy)-3-oxoprop-1-enyl)-7-(4-nitrophenoxy)carbonyloxy)-2,3-dihydrobenzofuran-3-carboxylate ***(5)***

To a stirred solution of compound **4** (500 mg, 0.40 mmol), Et_3_N (121 mg, 1.20 mmol), 4-DMAP (49 mg, 40 μmol) in CH_2_Cl_2_ (10 mL), and solid 4-nitrophenyl chloroformate (81 mg, 40 μmol) were added in a portion at 0 °C, and the mixture was stirred at RT for 1 h under N_2_. The mixture was partitioned between EtOAc and aq. NaHCO_3_, and the organic layer was washed with brine, dried over MgSO_4_, and evaporated. The residue was purified by silica gel column chromatography (*n*-hexane/ EtOAc = 2/1) to provide the compound **5** (477 mg, 85% yield). White solid; [α]_D_^20^ = +39 (c 0.38, CHCl_3_); IR ν_max _(KBr, CHCl_3_): 3068.19, 3030.59, 2952, 2923.07, 2856.06, 1784.8, 1742.85, 1643.54, 1616.06, 1593.4, 1526.86, 1495.04, 1447.8, 1347.52, 1251.58, 1219.27, 1179.26, 1154.67, 1076.08, 1044.75, 1020.16, 949.288, 918.914, 860.096, 813.331, 751.138, 700.516; ^1^H-NMR (250 MHz, CDCl_3_) δ = ^1^H-NMR (250 MHz, CDCl_3_) ppm 3.0 (m, 4H), 3.6 (s, 6H), 4.4 (d, *J* = 4.4 Hz, 1H), 5.2 (m, 2H), 6.0 (d, *J* = 4.4 Hz, 1H), 6.2 (d, *J* = 16.1 Hz, 1H), 6.5 (m, 2H), 6.6–6.8 (m, 7H), 6.9 (d, *J* = 8.5 Hz, 1H), 7.1 (d, *J* = 8.5 Hz, 1H), 7.3 (m, 20H), 7.5 (m, 13H), 8.2 (d, *J* = 9.2 Hz, 2 H); ^13^C-NMR (63 MHz, CDCl_3_) δ; 36.73, 37.33, 52.38, 52.58, 56.23, 59.05, 65.43, 73.52, 74.25, 88.22, 105.73, 108.55, 108.63, 108.67, 109.71, 109.74, 109.21, 109.86, 115.68, 116.81, 116.83, 117.44, 119.23, 119.74, 120.12, 120.36, 121.78, 122.30, 122.71, 122.98, 125.42, 126.16, 126.26, 126.34, 126.39, 128.32, 128.38, 129.19, 129.28, 129.75, 130.80, 133.57, 135.47, 140.10, 140.35, 140.47, 145.73, 146.22, 146.37, 147.22, 147.43, 147.72, 147.75, 147.85, 149.97, 151.14, 155.33, 165.49, 169.30, 169.54, 170.11, 171.26; MALD-TOF-MS calcd. 1426.3679 for C_84_H_61_NO_20 _[M+Na]^+^, found: 1426.3844.

### 3.4. (2S,3S)-(R)-3-(2,2-Diphenylbenzo[d][1,3]dioxol-5-yl)-1-methoxy-1-oxopropan-2-yl) 7-(2,2-dimethyl-4-oxo-3,9,12,15-tetraoxa-5-azaoctadecan-18-ylcarbamoyloxy)-2-(2,2-diphenylbenzo [d] [1,3]dioxol-5-yl)-4-(E)-3-((R)-3-(2,2-diphenylbenzo[d][1,3]dioxol-5-yl)-1-methoxy-1-oxopropan-2-yloxy)-3-oxoprop-1-enyl)-2,3-dihydrobenzofuran-3-carboxylate ***(7)***

To a stirred solution of compound **5 **(158 mg, 110 μmol) in CH_2_Cl_2_ (5 mL), *N*-*t*-butoxycarbonyl-4-7-10-1,13-tridecanediamine (35 mg, 110 μmol) and Et_3_N (46 μL, 330 μmol) were added, and the mixture was stirred at RT for 15 min. The mixture was partitioned between EtOAc and water, and the organic layer was washed with brine, dried over MgSO_4_, and evaporated. The crude product was purified by silica gel column chromatography (CH_2_Cl_2_/MeOH = 30/1) to give ethylene glycol linked LAB compound **7** (108 mg, 62%). White solid; [α]_D_^20^ = +45 (c 0.19, CHCl_3_); IR ν_max_ (KBr, CHCl_3_) 3379.64, 3062.89, 3008.89, 2945.73, 2926.93, 2864.74, 1744.3, 1720.19, 1636.3, 1587.13, 1496.97, 1449.72, 1366.32, 1251.58, 1215.42, 1155.15, 1043.78, 1020.16, 951.69, 815.742, 757.405; ^1^H-NMR (250 MHz, CDCl_3_) ppm 1.42 (s, 9H), 1.62–1.89 (m, 4H), 2.82–3.11 (m, 4H), 3.13–3.24 (m, 2H), 3.33–3.49 (m, 4H), 3.50–3.70 (m, 16H), 4.33 (d, *J* = 4.42 Hz, 1H), 4.96 (br. s., 1H), 5.12–5.27 (m, 2H), 5.87(br. s., 1H), 5.94 (d, *J* = 4.42 Hz, 1H), 6.19 (d, *J* = 15.80 Hz, 1H), 6.47 (d, *J* = 7.90 Hz, 1H), 6.51 (d, *J* = 1.26 Hz, 1H), 6.64–6.80 (m, 7H), 6.91 (d, *J* = 8.53 Hz, 1H), 7.08 (d, *J* = 8.53 Hz, 1H), 7.28–7.42 (m, 18H), 7.50–7.66 (m, 13H); ^13^C-NMR (63 MHz, CDCl_3_) δ 28.54, 29.10, 29.65, 30.97, 36.73, 37.29, 38.69, 39.81, 52.11, 52.29, 56.25, 70.13, 70.42, 70.48, 76.49, 77.00, 77.20, 77.50, 87.33, 105.83, 108.38, 109.50, 109.62, 116.64, 117.02, 118.42, 119.12, 119.91, 122.37, 122.67, 124.23, 125.23, 126.10, 126.18, 128.11, 128.15, 128.90, 128.97, 129.13, 129.56, 133.84, 136.30, 140.01, 140.14, 140.26, 140.30, 141.54, 145.97, 146.12, 146.98, 147.17, 147.25, 147.50, 151.70, 153.36, 155.92, 165.54, 169.16, 169.93, 170.15; MALD-TOF-MS calcd. 1607.5721 for C_93_H_88_N_2_O_22 _ [M+Na]^+^, found: 1607.9409.

### 3.5. (R)-Methyl-6-(tert-butoxycarbonylamino)-2-(5-(dimethylamino)naphthalene-1-sulfonamido)hexanoate ***(13)***

To a stirred solution of compound **11** (100 mg, 0.34 mmol) in CH_2_Cl_2_ (5 mL), dansyl chloride **12** (91 mg, 0.034 mmol) and Et_3_N (167 μL, 1.2 mmol) was added, and the mixture was stirred at RT for 3 h. The mixture was partitioned between EtOAc and water, and the organic layer was washed with water, dried over MgSO_4_, and evaporated. The crude product was purified by silica gel column chromatography (*n*-hexane/EtOAc = 2/1) to produce sulfonamide **13** (166 mg, 99%). Green yellow solid; IR ν_max_ (KBr, CHCl_3_) 3386.39, 3288.04, 2967.91, 2944.77, 2856.06, 2841.6, 2785.67, 1753.94, 1739.48, 1692.23, 1575.56, 1515.78, 1455.03, 1333.53, 1265.07, 1163.83, 791.636, 754.995, 627.716, 570.826; ^1^H-NMR (250 MHz, CDCl_3_) ppm 1.11–1.30 (m, 4H), 1.44 (s, 9H), 1.52–1.65 (m, 2H), 2.88 (s, 8H), 3.29 (s, 3H), 3.77–3.98 (m, 1H), 4.39 (br. s., 1H), 5.48 (d, *J* = 9.00 Hz, 1H), 7.20 (d, *J* = 7.42 Hz, 1H) ,7.55 (dt, *J* = 19.94, 8.04 Hz, 2H), 8.23 (dd, *J* = 7.27, 1.11 Hz, 1H), 8.32 (d, *J* = 8.53 Hz, 1H), 8.54 (d, *J* = 8.37 Hz, 1H); ^13^C-NMR (63 MHz, CDCl_3_) ppm 21.97, 28.39, 29.16, 32.53, 45.36, 52.11, 55.74, 115.27, 118.94, 123.08, 128.37, 129.67, 129.73, 129.78, 130.66, 134.64, 151.89, 155.86, 171.68; MALD-TOF-MS calcd. 516.2139 for C_24_H_35_N_3_O_6_S [M+Na]^+^, found:516.2399.

### 3.6. (R)-Methyl-2-(5-(dimethylamino)naphthalene-1-sulfonamido)-6-(2-methoxy-4-(3-(trifluoromethyl)-3H-diazirin-3-yl)benzamido)hexanoate ***(14)***

To a stirred solution of compound **13** (81 mg, 0.16 mmol) in CH_2_Cl_2_ (3 mL), TFA (1 mL) was added, and the mixture was stirred at RT for 2 h. The mixture was evaporated with toluene to remove TFA. The crude mixture was dissolved in DMF. To a mixture, EDC (92 mg, 0.48 mmol), DMAP (20 mg, 0.16 mmol), and diazirine acid **10** (42 mg, 0.16 mmol) were added. The mixture was stirred for overnight. The solution was quenched with water. The mixture was extracted with CH_2_Cl_2, _washed with water, dried over MgSO_4_, and evaporated. The crude product was purified by silica gel column chromatography (*n*-hexane/EtOAc = 3/2) to give compound **14** (65 mg, 64%). Yellow-green solid; IR ν_max_ (KBr, CHCl_3_); 3401.23, 2926.25, 2854.87, 1742.25, 1646.96, 1611.78, 1543.16, 1503.48, 1460.10, 1384.19, 1305.31, 1258.78, 1212.83, 1160.81, 1030.16; ^1^H-NMR (250 MHz, CDCl_3_) δ ppm 1.37 (m, 4H), 1.59–1.74 (m, 2H), 2.85 (s, 6H), 3.18 (s, 3H), 3.23–3.37 (m, 2H), 3.81–3.93 (m, 1H), 3.98 (s, 3H), 5.63 (d, *J* = 8.85 Hz, 1H), 6.68 (s, 1H), 6.88 (d, *J* = 8.06 Hz, 1H), 7.16 (d, *J* = 7.58 Hz, 1H), 7.51 (dt, *J* = 11.57, 8.04 Hz, 2H) ,7.74 (br. s., 1H), 8.21 (d, *J* = 8.06 Hz, 2H), 8.29 (d, *J* = 8.69 Hz, 1H), 8.52 (d, *J* = 8.53 Hz, 1H); ^13^C-NMR (63 MHz, CDCl_3_) δ ppm 22.13, 28.56, 32.38, 39.11, 45.33, 52.05, 55.69, 56.19, 109.09, 115.17, 118.71, 119.13, 122.73, 123.02, 128.36, 129.55, 129.64, 130.63, 132.84, 133.42, 134.34, 151.79, 157.29, 164.09, 171.58; MALD-TOF-MS calcd. 608.2037 for C_29_H_32_F_3_N_5_O_6_S [M-N_2_+H]^+^, found: 607.9775.

### 3.7. (R)-2-(5-(Dimethylamino)naphthalene-1-sulfonamido)-6-(2-methoxy-4-(3-(trifluoromethyl)-3H-diazirin-3-yl)benzamido)hexanoic acid ***(15)***

To a stirred solution of compound **14** (60 mg, 0.093 mmol) in THF:H_2_O (1:1, 3 mL), NaOH (8 mg, 0.19 mmol) was added, and the mixture was stirred at RT overnight. The mixture was partitioned between EtOAc and water, and the aqueous layer was extracted with EtOAc, dried over MgSO_4_, and evaporated. The crude product was purified by short silica gel column chromatography (CH_2_Cl_2_/ MeOH = 5/1) to produce the acid **15** (46 mg, 80%). Yellow-green solid; IR ν_max_ (KBr, CHCl_3_); 3397.96, 2927.57, 2853.74, 1729.28, 1643.58, 1612.40, 1546.25, 1505.57, 1462.46, 1411.93, 1308.42, 1259.36, 1213.62, 1180.80, 1160.46, 1030.76; ^1^H-NMR (250 MHz, CDCl_3_) ppm 1.35–1.44 (m, 4H), 1.68 (m., 2H), 2.83 (s, 6H), 3.17–3.25 (m., 2H), 3.92 (s, 3H), 3.97 (m, 1H), 5.96 (br. s., 1H), 6.68 (s, 1H), 6.85 (d, *J* = 7.90 Hz, 1H), 7.13 (d, *J* = 7.11 Hz, 1H), 7.45 (q, *J* = 8.27 Hz, 2H), 7.73 (br. s., 1H), 8.09 (d, *J* = 7.90 Hz, 1H), 8.21 (d, *J* = 6.95 Hz, 1H), 8.33 (d, *J* = 8.37 Hz, 1H), 8.48 (d, *J* = 8.53 Hz, 1H); ^13^C-NMR (63 MHz, CDCl_3_) δ ppm 22.02, 28.59, 32.25, 39.42, 45.34, 55.66, 56.30, 109.47, 115.39, 119.34, 122.75, 123.10, 128.29, 129.43, 129.74, 129.83, 130.51, 132.87, 133.71, 135.02, 151.57, 157.53, 164.62; MALD-TOF-MS calcd. 616.1700 for C_28_H_30_F_3_N_5_O_6_S [M−N_2_+Na]^+^, found: 616.2435.

### 3.8. (2S,3S)-(R)-3-(3,4-Dihydroxyphenyl)-1-methoxy-1-oxopropan-2-yl) 2-(3,4-dihydroxyphenyl)-4-(E)-3-((R)-3-(3,4-dihydroxyphenyl)-1-methoxy-1-oxopropan-2-yloxy)-3-oxoprop-1-enyl)-7-(R)-7-(5-(dimethylamino)naphthalene-1-sulfonamido)-1-(2-methoxy-4-(3-(trifluoromethyl)-3H-diazirin-3-yl)phenyl)-1,8-dioxo-13,16,19-trioxa-2,9-diazadocosan-22-ylcarbamoyloxy)-2,3-dihydrobenzofuran-3-carboxylate ***(3)***

To a stirred solution of compound **7 **(101 mg, 0.06 mmol) in CH_2_Cl_2_ (5 mL), TFA (1 mL) was added, and the mixture was stirred at RT for 24 h. The mixture was evaporated with toluene to remove TFA. The crude mixture was dissolved in DMF (1 mL). To a mixture, EDC (37 mg, 0.19 mmol), DMAP (7 mg, 0.06 mmol), and the acid **15** (40 mg, 0.06 mmol) were added and stirred overnight. The crude product was purified by silica gel column chromatography (CH_2_Cl_2_/MeOH = 10/1 to 5/1) to give the target compound **3** (15 mg, 16%). Amorphous yellow-green solid; [α]_D_^20^ = +24 (c 0.1, Methanol); IR ν_max_ (KBr, CHCl_3_); 3384.46, 2928.38, 1739.48, 1655.59, 1530.24, 1439.6, 1260.25, 1159.9; ^1^H-NMR (500 MHz, MeOD) ppm 1.48–1.57 (m, 5H), 1.78–1.80 (m, 4H), 2.01–2.04 (m, 1H), 2.83–2.86 (m, 6H), 2.94–3.04 (m, 6H), 3.41–3.58 (m, 16H), 3.65 (s, 3H), 3.67 (s, 3H), 3.92 (s, 3H), 3.95 (s, 1H), 4.38 (d, *J* = 4.58 Hz, 1H), 5.11–5.22 (m, 2H), 5.88 (d, *J* = 4.58 Hz, 1H), 6.31–6.39 (m, 1H), 6.50–6.82 (m, 9H), 6.97 (d, *J* = 7.90 Hz, 1H), 7.10 (d, *J* = 8.37 Hz, 1H), 7.21–7.29 (m, 2H), 7.50–7.62 (m, 4H), 7.88 (d, *J* = 8.25 Hz, 1H), 8.20 (d, *J* = 6.87 Hz, 1H), 8.36 (d, *J* = 8.71 Hz, 1H), 8.52 (d, *J* = 8.25 Hz, 1H); ^13^C-NMR (125 MHz, MeOD) ppm 23.97, 29.50, 29.88, 30.10, 30.75, 30.94, 33.25, 33.57, 37.13, 37.57, 37.92, 39.67, 40.56, 46.01, 48.52, 48.74, 48.95, 49.36, 49.58, 49.79, 52.89, 53.06, 56.98, 57.61, 58.17, 64.53, 69.81, 70.95, 71.30, 71.57, 71.97, 74.01, 75.11, 75.96, 89.24, 110.71, 113.45, 116.48, 116.64, 117.46, 117.59, 118.51, 119.61, 120.11, 120.71, 120.94, 121.88, 122.08, 124.48, 125.43, 125.57, 127.33, 128.63, 128.80, 129.49, 129.69, 130.84, 130.93, 131.08, 131.17, 131.23, 131.66, 132.89, 134.41, 136.72, 137.73, 142.91, 145.37, 145.53, 146.17, 146.34, 146.80, 147.06, 153.34, 156.31, 159.11, 167.08, 167.51, 171.29, 171.86, 172.21, 173.91; MALD-TOF-MS calcd. 1590.5020 for C_77_H_84_F_3_N_5_O_25_S [M-N_2_+Na]^+^, found: 1590.8215; HR(FAB) MS calcd. 1596.5268 for C_77_H_85_F_3_N_7_O_25_S [M+H]^+^, found: 1596.5226.

### 3.9. (2S,3S)-((R)-3-(3,4-Dihydroxyphenyl)-1-methoxy-1-oxopropan-2-yl) 2-(3,4-dihydroxyphenyl)-4-((E)-3-((R)-3-(3,4-dihydroxyphenyl)-1-methoxy-1-oxopropan-2-yloxy)-3-oxoprop-1-enyl)-7-((7R)-7-(5-(dimethylamino)naphthalene-1-sulfonamido)-1-(2-methoxy-4-(2,2,2-trifluoro-1-methoxyethyl)phenyl)-1,8-dioxo-13,16,19-trioxa-2,9-diazadocosan-22-ylcarbamoyloxy)-2,3-dihydrobenzofuran-3-carboxylate ***(16)***

A stirred 1 mM solution of compound **3** (1.8 mg, 1.13 mmol) in methanol (1.13 mL) was irradiated at 365 nm with a UV lamp (8 W, Waldmann, type 600352) at a distance of 1 cm for 1 h. The reaction mixture was concentrated at reduced pressure and the resulting product was characterized by UPLC-Q-TOF Mass analyzer. UPLC Q-TOF MS calcd. 1622.5283 for C_78_H_88_F_3_N_5_O_26_SNa [M+Na]^+^, found: 1622.8319.

## 4. Conclusions

In summary, we have described the design, synthesis and photochemical evaluation of a novel DLB photochemical probe **3** with a dansyl fluorescent tag. The major reaction included an amide formation of diazirine and dansyl linked acid **15** and DLB amine derivatives **7**. The DLB chemical probe **3** exhibited excitation at 335 nm and emission at 502 nm in aqueous solution. The Chemical probe **3** could be a valuable bioprobe for investigating ligand-protein interactions. Further studies to identify target proteins using this probe are now in progress and results will be published in due course.

## Supplementary Materials

Supplementary materials can be accessed on: http://www.mdpi.com/1420-3049/16/12/9886/s1.
